# Patient safety practices in an innovative digital landscape: an interview study on triage in Swedish digital primary care

**DOI:** 10.1080/02813432.2026.2648071

**Published:** 2026-03-23

**Authors:** Anna Gyberg, Jens Nygren, Petra Svedberg, Elin Siira

**Affiliations:** School of Health and Welfare, Halmstad University, Halmstad, Sweden

**Keywords:** Digital triage, healthcare professional, interview study, patient safety, primary care, qualitative content analysis

## Abstract

**Background:**

With rapid technological development and new triage systems in primary healthcare, care complexity is increasing, reshaping the landscape of patient safety. Despite their growing integration, little is known about how healthcare professionals safeguard patient safety during triage. Therefore, this study aimed to explore how healthcare professionals manage patient safety during the triage process in digital primary care.

**Method:**

An inductive and explorative research design was employed, using qualitative content analysis to analyse interviews with ten participants. The participants were healthcare professionals experienced with various triage systems.

**Results:**

Three themes were identified: acting as a fine-tuned instrument to avoid mistriage, resolving technical interruptions to prevent triage delays, and mitigating organisational disruptions to ensure adequate assessment. These themes reflected the participants’ continuous problem-solving efforts to maintain patient safety throughout the triage process.

**Conclusion:**

The findings suggest that triage systems may be overly simplified, shifting greater responsibility to healthcare professionals, who must interpret system outputs and manage the broader complexities of primary care triage. We argue that triage safety emerges from the interaction between an understanding of the healthcare system’s contextual capacity, broad medical knowledge, and the interpersonal skills needed to recognise and respond to each patient’s unique situation.

**Clinical implications:**

Clinically, it is essential to define in advance the boundaries and procedures of the triage process, the mode of digital integration, and the system’s intended purpose. Specifying these elements helps ensure that the digital tool supports rather than complicates clinical work and helps establish the preconditions necessary for patient safety.

## Background

Globally, including in Sweden, primary care is increasingly being digitalised. The rise of digital approaches, such as remote triage, raises important questions about the safety of these new modalities and how healthcare professionals address and manage these concerns. One of the challenges in determining the direction of care for each patient in primary care is that it serves the entire population and encounters a wide range of symptom presentations [1]. This initial assessment, triage, refers to the process by which patients are prioritised based on the level of care they require, given the inherent limitations of existing resources. In such scenarios, healthcare professionals conduct a brief assessment of the patient’s medical condition with the assistance of a set of criteria to determine the level of urgency or the appropriate treatment [2]. As many patients are seen in the early stages of disease progression in primary care, there are fewer cues to consider during the initial reasoning about the patients’ medical condition. Thus, healthcare professionals must also be particularly responsive to contextual behaviour and environmental cues [3]. Early stages of disease, which are commonly encountered in primary care, must be assessed with a higher level of uncertainty than in the later stages, where more advanced and differentiated presentations can be observed [3]. The accuracy of this initial assessment is critical to patient safety and the risk of adverse events, as early assessments often persist, for example, through confirmation bias, making it difficult to reconsider alternative diagnosis and treatment options once a preliminary hypothesis has been formed [4].

The patient safety work in this study is viewed to continue throughout the entire triage process, from the moment patients encounter the primary care system until the urgency of the patient’s condition and appropriate treatment are determined. Although adverse events, which are avoidable injuries and suffering caused by medical management [5], are rare in primary care, the burden of unsafe care is still too high [6]. In the United Kingdom, primary care, significant preventable harm was estimated to be 35.6 per 100,000 patient years [7]. More specifically related to triage, these adverse events involve problems with diagnosis (60.8%), such as an incorrect or delayed initial diagnosis, as well as delayed and missed referrals (10.8%) [7]. Patient calls, which are not being properly forwarded, have been reported to result in 3% of patients experiencing harm and 26% prolonged pain and discomfort [8]. Mistriaging, or incorrect assignment to a higher or lower level of care, is accordingly known to have the potential to worsen a patient’s health condition. Additionally, overestimation of the illness can result in the utilisation of already limited resources, potentially denying them to patients in greater need. Overestimation may also harm patients through unneeded examinations and treatments [2]. Thus, the accurate assessment of urgency, along with correct diagnosis, appropriate investigation, and treatment, is one of the greatest patient safety concerns during the triage process.

To triage patients accurately has been referred to as a process that requires a broad expertise and solid experience due to its’ challenging and complex nature, involving rapid analysis of the patient’s situation and planning of the next step of action as well as communication about the patient’s condition and expectations with the patient and other staff members [9,10]. However, with limited resources and the ambition to provide a more accessible care, several options have been developed for patients to connect with primary care in situations that can be managed just as well outside of the institutional setting. Consequently, and with the lack of a gold standard for triage [11], there are today multiple pathways through which patients can access primary care.

These pathways include, e.g. telephone triage [11], video consultations [12], digital self-service tools [13], telemedicine triage based on artificial intelligence (AI) *via* smartphone applications [14,15], automated patient interviewing software [16], and more. It has been demonstrated that there are numerous advantages, including the provision of rule-based workload relief through the utilisation of, for example, automated patient history-taking software [17] or AI virtual assistance for patients to self-assess their symptoms and decide on the need and appropriate level of care [18]. The advent of multiple novel technologies invariably gives rise to novel insights, as well as consequential challenges that subsequently raise patient safety concerns.

For example, Krylova et al. [13] addressed that several decision-tree models, that is, questionnaire-based triage systems with predefined rules for determining care levels, rely on patients completing the questionnaires. Furthermore, the potential of machine learning to predict the outcomes of incomplete triage interviews had a linear correlation between the prediction accuracy and the degree of completeness. Other presented patient safety concerns are that digital tools designed to generate a set of differential diagnoses and clinical triage advice based on symptoms and biodata that patients enter *via*, e.g. an application, vary across medical conditions and generally perform poorly [19]. Also, the authors stressed that a safety-first approach, when using probability-based clinical decision-making tools, needs to consider that low-risk symptoms might mask life-threatening conditions. These studies provide examples of the interplay between technology, human behaviour, and the dynamic presentation of medical conditions and their symptoms, demonstrating how easily patient safety can become uncertain and unstable.

The patient safety challenges presented above illustrate how difficult it is to predict patient safety risks and to design and predefine safe components of the triage process. This aligns with the perspective on patient safety adopted in this study, which holds that patient safety does not reside in a specific device or individual [20]. Instead, patient safety is understood as socially constructed through real-world practices, emerging in the interactions between people and technical components within a system. It is contingent upon how involved actors, in this case, healthcare professionals, interpret unfolding situations at the point of care [21,22]. As such, patient safety cannot be easily ensured through simplified or standardised processes and routines [21]. Thus, patient safety is viewed to be the result of actions and non-actions based on how situations are perceived as safe or unsafe. Cook et al. [23] use the terms ‘gap’ and ‘discontinuity’ to describe situations of unsafe care in any healthcare setting that pose a risk to patient safety. These gaps can result from various interruptions in care, such as events requiring a departure from routine procedures, misinformation, or insufficient resources. Healthcare professionals normally identify and resolve these discontinuities, either consciously or unconsciously. However, these resolved unsafe situations can be subtle and difficult to identify. They can also mask significant flaws in the system, especially during organisational or technical changes.

The introduction of novel technologies in healthcare settings contributes to the increasing complexity of care, thereby placing greater demands on healthcare professionals and their capacity for adaptability [24]. Adopting a situational-related approach to patient safety in this study enables an exploration of healthcare as a complex system, focusing on how patient safety is enacted in clinical practice and the possibilities and challenges this generates [21]. Because no gold standard for correct triage exists [11] and the situation-specific nature of patient safety, the exploration of patient safety work is challenging. However, Cook et al. [23] suggest that substantial and useful knowledge about patient safety can be obtained by exploring the various types of unsafe situations healthcare professionals encounter during technical changes, as well as how they anticipate, identify, and resolve unsafe situations. This approach sheds light on the complexity of patient safety risks and how healthcare professionals apply their expertise and capabilities in various situations in everyday real-world practice to provide safe care.

The developments in technology, including the introduction of triage solutions with the potential to enhance or even replace certain aspects of the triage process, have resulted in a significant transformation of the patient safety landscape. Therefore, the aim of this study was to explore how healthcare professionals manage patient safety during the triage process in digital primary care.

## Method

### Study design

This study employed an inductive qualitative design. Semi-structured interviews were conducted to explore healthcare professionals’ experiences, and the data were analysed using qualitative content analysis as described by Graneheim and Lundman [25]. The study is reported in accordance with the Consolidated Criteria for Reporting Qualitative Research (COREQ) [26] to ensure clear and comprehensive reporting.

### Setting

This study was conducted in a digital primary care setting as part of a public primary healthcare organisation with over 5,000 employees in a Swedish region. The digital primary care allowed patients to schedule digital appointments *via* web or mobile application. Designed to handle less complex medical issues, the service operated from 7 am to 11 pm, seven days a week and managed approximately 50,000 digital consultations annually. Patients who sought care outside these hours could submit their information, but their cases were not processed until the service reopened. It was staffed by general practitioners, nurses, and psychologists. While the digital primary care functioned as an independent unit within the primary healthcare organisation, many healthcare professionals working in the service were also employed at physical primary care centres within the same organisation.

The digital primary care was characterised by a strong focus on innovation and continuous development, particularly through the testing and implementation of new digital solutions. As part of this approach, the digital primary care had trialled several digital applications to support the triage of patients to manage patient inflow, ranging from advanced AI-driven systems to simpler digital forms. During the past two years, the interviewed healthcare professionals had used at least two of three different digital applications. These applications were designed to collect patient information, assess medical conditions, and direct patients to appropriate levels of care. One of these applications was implemented but discontinued shortly thereafter due to concerns related to patient safety. Despite its brief use, this example highlights critical issues surrounding patient safety. All three applications were questionnaire-based systems, followed by video consultations for further assessment of the patients’ medical condition. One employed AI-driven questioning to support differential diagnosis; another utilised a deterministic, adaptive questioning format for the same purpose; and the third consisted of a static questionnaire without any diagnostic functionality. With such a broad experience of digital systems in an innovative digital care environment, the healthcare professional offered valuable perspectives on how patient safety was managed, including important lessons from the discontinuation of one application due to safety concerns.

### Participants and data collection

The recruitment process was facilitated through collaboration with the manager of the digital primary care, who was asked to provide contact details for eleven individuals meeting the following inclusion criteria: (1) being employed as healthcare personnel or in a managerial and/or strategic role within the primary care organisation, and (2) having experience with one or both of two distinct digital applications used over the past year to collect patient information, assess medical conditions, and direct patients to the appropriate level of care. To ensure variation, the manager was asked to select participants representing a range of professional roles within the organisation and subsequently provided contact details for eleven members of the digital primary care staff. All individuals were contacted and informed about the study. All participants met the eligibility criteria; nine agreed to take part in an individual interview, while two declined. A pilot interview was conducted before the main data collection to test the interview questions. As no modifications to the interview guide were required, and with the participant’s consent, the pilot interview was included in the final analysis. This resulted in a total of ten interviews.

The participants, eight female and two male, held diverse roles, including clinical (*n* = 7) and administrative (*n* = 3) healthcare professionals: two general practitioners, three registered nurses, two psychologists, two digital health specialists, and one quality improvement manager. Collectively, the participants had between 1.5 and 21 years of experience at their current workplace and between 6 and 40 years of total professional experience. All participants occupied roles within the digital service that entailed involvement in the use, management, or oversight of the development, implementation, and operation of the two digital applications designed to handle patient inflow.

The individual interviews were conducted *via* video communication in January 2024. A PhD student, trained and experienced in qualitative interview techniques, served as the interviewer. There was no prior relationship between the interviewer and the participants. A semi-structured interview format [27] was employed, guided by an interview protocol consisting of open-ended questions. These questions explored aspects of patient safety in relation to the development, implementation, and use of the two digital applications, aiming to elicit a nuanced understanding of the participants’ experiences. To encourage elaboration and deepen the narratives, the interviewer used follow-up prompts such as, ‘Could you elaborate on that?’ The interviews lasted between 36 and 62 min (mean = 50), were audio recorded, and subsequently transcribed verbatim.

### Data analysis

The analysis was guided by qualitative content analysis. This method was employed as it aligns with the inductive approach of this study, allowing for the systematic categorisation process of interview text and the interpretation of patterns and their meaning [28]. Both manifest and latent content of the text were analysed, that is, both what was said verbatim and the underlying meaning. The analysis of the manifest content allowed the researchers to remain close to the text, while the subsequent latent analysis enabled a more interpretive approach. Together, these analytical levels contributed to an analysis encompassing both concrete and abstract dimensions. Interpreting the underlying meanings in this way supported the identification of themes at different levels, while simultaneously remaining close to the empirical data and avoiding overly extensive interpretation [28]. The initial phase of the analysis involved a comprehensive reading of the interview transcripts to gain an overall understanding of the content. The following step entailed the decontextualisation of the content, with a focus on identifying meaning units. These units generally comprised a sentence, a cluster of sentences, or paragraphs that conveyed a shared essential meaning. If feasible, these units were then condensed in a manner that essentially captured their meaning. The meaning units were subsequently coded and also recoded, particularly during the stage of the analysis, where meaning units were iteratively compared and grouped based on conceptual similarity. Following de-contextualisation, the data were re-contextualised as themes began to emerge. Memos were drafted to record ideas, track the analysis process, and facilitate reflexivity throughout the analysis process. See Table 1 for an illustration of the analysis process. The first author (AG) conducted the majority of the analysis, from coding and recoding meaning units to the iterative grouping of units based on conceptual similarity, while the last author (ES) challenged interpretive decisions and provided reflective input to the analysis. AG and ES drafted the findings, and the remaining two authors (JN, PS) critically reviewed them, contributing their expertise to ensure clarity and alignment with the aim of the study.

**Table 1.** Illustration of the analysis process.

Citations	Condensed meaning units	Codes	Themes
*If you answer one, you end up there, if you answer zero, you end up there. There is no grey area, there is no empirical and clinical experience that can assess this grey area that often arises when sorting. It’s more like ones and zeros, and that’s what doesn’t work.* [Participant 1]	There is no grey area, nor is there room for empirical or clinical judgment to address the nuances that often arise in practice.	Governing the authenticity of data	Acting as a fine-tuned instrument to avoid mistriage
*Yes, and some patients don’t get a box to tick when they book their meeting. Because they have to approve the audio and video, and sometimes the patient hasn’t got these boxes, and you have to call the patient instead.* [Participant 2]	As the required options to confirm audio and video settings when booking sometimes are missing, staff must contact the patients by phone instead.	Resolving technical interruptions	Resolving technical interruptions to avoid triage delays
*Before, when you applied for mental health, you could choose whether you wanted to see a psychologist or a doctor. They took that away.* (…) *But it has become more complicated. And I’m not satisfied with the fact that it’s not possible, that patients with mental illness often end up in the wrong place* (seeing the doctor instead of the psychologist). [Participant 5 ]	The option to choose between a psychologist or a doctor for mental health was removed. This has made the process more complicated, and patients with mental illness often end up in the wrong place.	Steered to the wrong professional category	Managing organisational disruptions to the triage process to ensure adequate assessment

### Ethics approval and consent to participate

This study followed ethical principles according to the Declaration of Helsinki [29]. No ethical approval from the Swedish Ethical Review Authority was deemed necessary based on thorough ethical reflection within the research team. Although the interviews explored the experiences of healthcare professionals regarding patient safety, the focus remained on their work within the digital triage process and did not include sensitive personal data. Additionally, the performance of the interviews and the content of the research topic were determined to pose no risk to the participants. All participants were informed, both orally and in writing, about the aim of the study and of their right to withdraw without having to provide a reason. They were also informed that all data was handled in accordance with the General Data Protection Regulation (GDPR) [30]. All participants consented to participate in the study before being interviewed.

## Results

The participants operated within a digital environment marked by a strong emphasis on innovation and ongoing development, including the trial and use of various digital applications for triage. It became evident that participants drew on their accumulated experience with different triage systems to interpret and manage both prerequisites for patient safety and patient safety risks. From the participants’ perspective, triage was a process that comprised not only a set of criteria for understanding and acting on symptoms, but also a wide-ranging repertoire of knowledge carried by healthcare professionals that took additional values into account for safe and timely triage.

The triage process in the digital primary care setting described by the participants began when a patient chose to seek care and accessed the digital platform. The patient was then guided through a standardised set of automated questions related to their symptoms. Based on the responses, the patient was offered the option to book a short video meeting with a healthcare professional. The triage process concluded during the video meeting, where the healthcare professional conducted a medical assessment. During this meeting, the healthcare professional assessed the patient’s medical condition and determined whether the patient had reached the appropriate level of care or needed to be referred to another healthcare provider.

The analysis of how healthcare professionals manage patient safety during digital triage in primary care revealed that the participants: *acted as a fine-tuned instrument to avoid mistriage*, *resolved technical interruptions to avoid triage delay* and *mitigated organisational disruptions to ensure adequate assessment.* Despite these dedicated efforts, lasting solutions were often out of reach, resulting in continuous, temporary, and recurring problem-solving daily, as described in the following three themes.

### Acting as a fine-tuned instrument to avoid mistriage

To ensure a solid assessment, the participants relied on their capacity to read the patient and adapt their questioning style according to the patient’s narrative, expression and situation, which was enabled during a subsequent video call after the patients had answered a set of questions. This included timing and being respectful and perceptive, e.g. by rephrasing questions in instances when needing more details or when the patient did not appear to comprehend the inquiry. Essentially, the participants’ actions were driven by a sense of confidence in their ability to comprehend the patients’ medical condition better than the digital systems and subsequently assumed the responsibility for ensuring patient safety and resolving potential hazardous triage. This way, the healthcare professionals relied on and valued themselves as a fine-tuned instrument to create a well-supported foundation to be able to make an appropriate assessment of the patient’s condition and situation.

The participants emphasised how they saw a risk for mistriaging when a digital system posed questions without adapting to the patient or without ensuring that the patient understood the questions. They described how predetermined questions and definitions that they often used could be interpreted in various ways depending on the patient’s situation and perceived needs. As one participant described:

You have to see it from the patient’s perspective; otherwise, you don’t really understand what it’s like. That’s why questions like these have come up—they feel very irrelevant and strange. For example, a question like, ‘Do you think you have Alzheimer’s?’ Where does that even come from? Why ask such questions? These are ordinary people who may not even know what this disease is. They shouldn’t be the ones to decide if they have it or not. There were so many odd questions that made me think, ‘Oh my God.’ [Participant 8]

Questions posed by digital systems to collect information about the patients’ condition were criticised for being too many, irrelevant, or difficult to understand or not applicable to the patient’s condition. One participant expressed:

It’s pre-programmed. […] it’s not all the pieces in the puzzle, but it’s just like some sporadic pieces here and there, and then they’re thrown in, and then you are expected to still be able to cover the whole puzzle. [Participant 1]

Simultaneously, the participants found themselves having more questions than the digital systems offered answers to. Additionally, the participants addressed the problem with determinism being irreversible, that is, that the patients were not allowed to go back and change their responses. Subsequently, deterministic questions resulted in the formulation of diagnostic hypotheses that initially could have been derived from an inappropriate interpretation of the question’s meaning, which they consequently could not rely on. At the same time, the suggested diagnostic hypotheses could also provide insights into ways of thinking that the participants themselves had overlooked. When receiving a summary about the patients’ condition, it could prove challenging to discern which words were uttered by patients and which were pre-constructed within the system. Thus, in the encounter with the patients, they used their capacity as fine-tuned instruments to collect information they could rely on to be able to assess the patients’ condition. One participant emphasised that in their professional role, they could not outsource responsibility to digital systems:

It’s still our responsibility to find out whether it’s really true that this is safe to use or not. Because at the end of the day, it’s our responsibility as healthcare providers, even if someone else has decided and told us to use it [the digital triage system]. [Participant 1]

Participants’ sense of responsibility for patient safety in the triage process extended well beyond their immediate clinical encounters. They did not regard the digital systems as providing comprehensive medical histories or established clinical assessments, but rather as limited tools within a broader clinical and organisational context. Importantly, their engagement with patient safety also encompassed organisational dimensions, such as evaluating which systems to adopt and understanding how these systems shaped triage practices over time. Participants were actively involved in the development and refinement of digital triage, including the testing of new systems and assessing their usefulness in everyday clinical work. In this way, responsibility for patient safety was understood not only as a clinical obligation but also as a systemic concern embedded in organisational decision-making.

In their everyday clinical encounters, the participants utilised the digital systems in ways they saw as useful. They tended to maintain a preference for preparing their meetings with the patients by reading previous medical records and relying on the patients’ narratives during the appointments rather than relying on the systems. With appointments scheduled within strict time frames, opportunities to build trusting relationships necessary for accurate assessments were constrained, risking that interactions were reduced to more ‘*mechanical dialogues*’ [Participant 5]. Thus, while the participants sought to use themselves as fine-tuned instruments in triage, this was not always possible in practice.

### Resolving technical interruptions to avoid triage delays

The participants frequently mentioned the technical challenges when reaching the phase of the triage process when they had the actual meeting with the patients. Mainly, the interruptions consisted of technical interruptions and technical support taking the centre stage in the conversation with the patients. Resolving technical interruptions was a continuous task that healthcare professionals engaged in daily, not only during patient encounters, but also by addressing the issue at an organisational level. This included actions such as submitting incident reports and raising concerns through internal channels. The management team also held recurring meetings to manage technical problems and maintained regular contact with the developers of the digital systems. Furthermore, not all patients demonstrated the capacity to manage the digital services, resulting in healthcare professionals spending a considerable amount of time guiding the patients in how to connect to the meeting online. As one participant described:

It’s not easy for everyone. Very often, you need to instruct them on how to do it. And if you call back, if they can’t connect, I call back and say ‘well, let’s try again’ and so on [Participant 4]

In this context, participants emphasised that some patients were unable to utilise the digital service to begin with, due to digital exclusion, including the lack of multilingual options and the presence of cognitive impairment or other functional inabilities that may impede access to digital platforms. From the participants’ perspective, digital triage inherently excluded certain patient groups. However, they noted that some of these barriers could be mitigated when patients were able to select a healthcare professional with whom they had an established relationship, often due to long-term illness, or a provider who was bilingual and could communicate in the patient’s preferred language. Such continuity in care not only facilitated the triage process but also enhanced its quality, as mutual understanding could be more readily achieved through an existing relationship or shared language. Such functions were, from their experience, provided by certain digital triage systems but not others.

When having the meeting online with the patients, insufficient quality of the audio and video could also disturb the triage process of creating an authentic basis for assessment. As one participant described:

I think there is a concern among us as doctors and nurses, and psychologists, that you miss information because the sound may not work, and you miss what the patient says. It stutters a bit and so on, so I think we have not, overall, we have not been very satisfied [Participant 9]

Thus, the inability to see or hear patients constituted a recurrent issue, rendering it challenging to engage in a productive conversation. Often, the healthcare professionals had to ensure the functionality of the audio and sound equipment to facilitate the meeting. This took time from the patient meeting, thus diverting healthcare professionals from their brief assessment of patient urgency and treatment options, thereby delaying the triage process. A prolonged disruption in the meeting occasionally led to patients departing from the meeting, subsequently necessitating their self-re-triage within the healthcare system.

### Managing organisational disruption to the triage process to ensure adequate assessment

During the triage process, specifically before its completion through an appropriate medical assessment, it became evident that patients were sometimes incorrectly allocated: externally, by being directed to digital primary care from physical primary care, and internally, by being assigned to specific professional categories, based on organisational factors. This potentially led to a lack of adequate assessment, resulting in postponed access to appropriate care and an increased risk of prolonged suffering or deterioration in the patient’s health.

One such organisational factor was the great number of patients seeking care in the physical primary care, which exerted considerable pressure on healthcare professionals, leading to a great number of referrals to digital primary care even when physical examination or treatment was needed. The participants distinguished between the level of care provided in physical and digital primary care settings. They acknowledged a distinction in terms of available resources for assessing a patient’s condition, and they were aware of the limitations inherent in digital triage, such as the inability to obtain a full physical impression of the patient during a video meeting. Consequently, this became an important factor to consider when interacting with patients in the digital setting:

Yes, and I think maybe it’s not so much because of this system or previous systems, but it’s the high pressure of care in general that many people are seeking care and that it becomes overcrowded at our primary care centres. And the stress means that ‘yes, but we refer to online’, without really listening to the fact that patients need to come to the physical primary care centre for the sake of the patients’ safety in some cases. [Participant 2]

As the quote describes, referrals were sometimes made without a comprehensive clinical assessment, leading to stressful work situations. In addition to organisational factors, such as high patient volumes and time constraints, patients were also routinely encouraged to seek care *via* digital primary care, even when initially contacting physical primary care units. For instance, callers to physical primary care were met with an automated voice message informing them of the option to seek medical care online. However, this message was sometimes interpreted by patients as an exhortation rather than a voluntary option, potentially leading to a systematically misdirected care-seeking behaviour.

To address the issue of incorrect allocation, healthcare professionals often redirected patients to physical primary care during the video meeting. As a result, participants frequently found themselves re-referring or redirecting patients. This led to a recurring cycle of re-referrals, which in turn significantly delayed the process of reaching an appropriate triage. Simultaneously, the interviews revealed that the number of patients seeking digital primary care had also increased recently, which had compelled general practitioners to take on extra shifts to manage this inflow of patients.

Another organisational factor that could contribute to delays in the triage process was the potentially misaligned referral of patients to specific professional categories. It was recognised that the digital systems used in digital primary care, regardless of whether they were explicitly perceived as triage tools, had a significant influence on how patients were directed within the organisation. This was particularly evident in the booking process, where patients were often assigned to a professional category based on predefined configurations within the digital platform. For instance, this resulted in patients being automatically scheduled for an appointment with a general practitioner, unless they had explicitly indicated a preference for another type of healthcare category. This was exemplified by a nurse who described a noticeable shift in workload, linked to the way booking of meetings was configured in the digital primary care platform:

Nurses were fully booked quite often before. And then when the patients weren’t triaged in that way because there’s no AI now (previous triage system), they’re very often directed to a doctor. So the doctors are fully booked. (…) So we have to go out at short notice and ask doctors throughout the day, ‘Is it possible for you to take extra shifts today?’ Because the doctor’s appointments run out very quickly because the patients are directed via the forms there and not to the nurse. [Participant 9]

Healthcare professionals did not necessarily redirect patients between professional categories at this stage. Instead, they managed the consequences of this initial booking by adjusting staffing levels, which in this case involved increasing the number of general practitioners. Participants reported having experience with various system configurations that influenced the flow of patients to different categories of healthcare professionals. This posed a challenge to the triage process, as certain professional categories became overloaded while others were underutilised. For example, psychologists were not authorised to prescribe medication or issue medical certificates, which meant that patients presenting with psychological concerns were frequently referred to general practitioners instead. This sometimes led to missed opportunities for potentially valuable consultations with psychologists. On the other hand, when patients were directed to psychologists despite only requiring a prescription, the psychologists were obliged to redirect them to general practitioners, causing misallocation of professional resources, risking that patients with less appropriate care needs displaced others with more urgent needs to see a psychologist. Taken together, organisational factors disrupted the triage process, necessitating considerable additional effort from healthcare professionals to ensure that patients were appropriately directed for an adequate assessment to finalise the triage process.

## Discussion

The exploration of how healthcare professionals manage patient safety during the triage process in digital primary care revealed that this involved continuous problem-solving on a daily basis. It required healthcare professionals to draw on their capacity to read and adapt to the patient’s narrative, address persistent technical challenges, and re-refer patients. It was evident that healthcare professionals undertook substantial efforts to ensure that patients receive adequate assessment, thereby facilitating the completion of the triage process and mitigating the risks of mistriage and triage delays.

The findings of this study suggest that digital systems used in triage may have limitations in representing the full scope of the process, as also suggested by previous research. For instance, limited possibilities to collect sufficient patient information [31] and the inaccuracy or lack of clarity in symptom checker assessment reports prompting additional questioning [32] adding patient safety responsibility onto healthcare professionals—not only to interpret the outputs generated by these digital tools, but also to manage the broader scope inherent in triage processes.

Taking responsibility for patient safety meant that healthcare professionals assumed additional tasks, such as interpreting and supplementing the outputs generated by the digital triage systems. This pronounced sense of responsibility for patient safety aligns with the findings of Elgin and Elgin [33], who observed a sense of hesitation among healthcare professionals, stemming from the recognition of significant risks associated with relying too heavily on decision-support systems whose limitations and potential biases are not fully understood. Thus, healthcare professionals in the study by Elgin and Elgin [33] expressed a strong need to develop methods for verifying and validating information, particularly in high-stakes situations involving decisions with direct implications for patient outcomes. Our findings indicated that such methods or strategies also guided healthcare professionals’ actions by enabling them to either utilise information from triage systems or dismiss it when considered unreliable or insufficient.

Furthermore, healthcare professionals devoted considerable effort to managing technical issues related to the triage systems. Continuous adaptation to the realities of everyday clinical practice was necessary to ensure adequate patient assessment. However, these adjustments rarely provided permanent solutions. Instead, healthcare professionals were often required to spend unwanted time troubleshooting technical problems, which not only delayed the triage process but also risked patients leaving the encounter without being assessed. Such additional work has previously been shown to increase the complexity of healthcare professionals’ tasks and is often described as invisible or ‘silent’ work that remains unrecognised within the organisation [34]. The same authors also highlight how this added workload encroaches upon the time available for direct patient care, a finding echoed in our study. Overall, this illustrates that ensuring patient safety in digital triage systems extends beyond the clinical encounter to include the continuous maintenance of technical systems.

These types of additional tasks to ensure patient safety may inevitably diverge from how tasks are ideally intended to be performed according to formal rules or standards [35]. Such divergences can both mitigate potential adverse events in certain situations and, paradoxically, contribute to unintended problems in others. When rules, standards, or guidelines are developed for digital triage systems, they are typically designed to promote safe, timely, and efficient care, as exemplified by several existing triage systems [14,36]. However, this is not always the outcome. By attending to ongoing interactions between healthcare professionals, patients, the organisations and technology as sites of safety production [22], our study illustrates how patient safety in digital triage emerges through healthcare professionals’ continuous efforts in everyday practice rather than simply through the utilisation of digital triage systems.

Moreover, there is a lack of clarity regarding what delineates ‘digital triage’ from other forms of triage. There are numerous types of digital triage used in different disciplines in healthcare [37]. Furthermore, the conceptual definition of digital triage is vague. For instance, in terms of telephone triage, digital triage is referred to as a ‘call handler or clinician using a digital triage tool’ [38], in other instances it is described as including online symptom checkers [39] or divided into either telephone or text-based triage [31]. Although we do not aim in this article to provide an overarching definition of digital triage, we argue that it is important for research to theorise around empirical phenomena. Such theorisation facilitates a clearer understanding of the concepts under study and their potential implications, as in this case, regarding patient safety and comparisons across studies of different digital triage technologies. We agree with previous research that digital triage is complex and that its outcomes may be influenced by multiple factors depending on the healthcare context [38] that, when implemented, will alter the ways healthcare professionals involved in the triage of patients work [39] and that it demands other skills than traditional physical face-to-face triage [31]. We also argue that in relation to digital triage, healthcare professionals’ management of patient safety should be understood as dependent on the interplay between a contextual understanding of the healthcare system’s capacity, comprehensive medical knowledge, and the interpersonal ability to understand and respond to each patient’s unique situation.

Compared to other clinical settings, such as emergency care, triage is less theoretically and empirically explored in primary care [40,41]. Our findings illustrate that triage can be a considerably more complex and far-reaching process than is suggested by prevailing definitions [2] and exceeds what current digital systems appear capable of handling. We suggest that triage should be seen as a complex and time-sensitive process which, as our findings illustrate, can be compromised in a digital primary care setting by overly simplified and standardised questions, technical malfunctions, or competing organisational priorities. Triage is not solely about gathering information or assigning diagnoses. Besides engaging with the patients account, it requires an in-depth understanding of the healthcare service itself, its resources, capacities, specialisations, and internal organisation, as well as an awareness that identical symptoms may signify different conditions depending on the context, including how they manifest, how frequently they occur, and under what circumstances. While this study focuses on the perspective of healthcare professionals, it also points to the importance of investigating the patient’s role as an active participant in safeguarding their own care within digital triage systems [22]. The findings highlight the complexity of the primary care triage process, particularly in relation to digital triage, underscoring the need for future research to address both empirical and theoretical aspects of digital triage to ensure safe and effective patient care.

### Methodological consideration

The relatively small number of interviews conducted constitutes a limitation of the study, as a larger sample might have provided a broader range of perspectives and further nuance. Data collection was concluded after 10 interviews due to several considerations. First, the sample included a diverse range of professional roles and experiences relevant to the research question, which contributed to a rich and varied dataset. Second, practical constraints such as the limited availability of participants working in high-demand healthcare roles also influenced the final number of interviews. Despite the relatively small sample size, the data obtained were considered sufficiently rich and nuanced to support a meaningful and credible qualitative analysis. Recruitment through the manager may have introduced some selection bias. However, the manager was well-positioned to identify potential participants with sufficient experience of the digital systems, ensuring that we could obtain data relevant to our research questions.

Examining triage as a process presents considerable challenges, as it is, in many respects intangible, and the influence of digital tools extends across multiple perspectives and multiple dimensions of healthcare professionals’ work. For example, this study did not include patients’ experiences of using digital triage tools, nor did it address the accuracy of triage decisions or subsequent care. However, as our patient safety perspective is grounded in the view that patient safety is supported by multiple actors [42], rather than solely by clinical staff, we included several categories of healthcare professionals, encompassing both clinicians and digital health specialists. Viewing all participants as resources in patient safety work enabled us to explore how they adapted to varying conditions and how procedures were adjusted to maintain patient safety from multiple perspectives. Overall, these considerations suggest that the transferability of the findings should be interpreted with caution. Nevertheless, in an era characterised by rapid and ongoing technological development, exploring these complexities is crucial for deepening understanding of real-world practices and how healthcare professionals navigate and adapt to these evolving conditions while upholding patient safety. To strengthen the evidence base, further studies exploring a wider range of perspectives on patient safety and digital triage systems are needed, particularly those of groups that are often overlooked, such as patients who do not complete their initiated care-seeking processes in digital primary care settings and healthcare professionals, including digital health specialists, as well as other healthcare professionals working at the interface between clinical practice and digital systems. Incorporating a broader diversity of perspectives may improve patient safety in triage by addressing blind spots in the current knowledge base.

## Conclusion

This study explored how healthcare professionals manage patient safety during the triage process in digital primary care. The findings indicate that triage is not merely a set of criteria for interpreting and acting upon symptoms, but rather a complex process encompassing a broad repertoire of professional knowledge that integrates additional values to ensure safe and timely decisions. These results highlight the multifaceted expertise required of healthcare professionals to maintain patient safety when using digital triage systems, which currently provide limited guidance on how to align with existing triage processes, placing the responsibility for interpretation and integration on the professionals themselves. As a clinical implication, it is essential to establish, before implementation, where the triage process begins and ends, how it is carried out in the specific clinical context, when and how the digital triage solution is intended to be integrated, and what it is designed to achieve. Clarifying these aspects appears to be key in determining whether the digital system will ultimately serve as a helpful tool or not.

## Data Availability

The data that support the findings of this study are available from the corresponding author, AG, upon reasonable request.

## AI-based tools and technologies

The AI tools ChatGPT and Grammarly were employed to support language refinement during the writing process. The authors retain full responsibility for all content.
